# Design and marketing features influencing choice of e-cigarettes and tobacco in the EU

**DOI:** 10.1093/eurpub/ckw109

**Published:** 2016-07-28

**Authors:** Anthony A. Laverty, Constantine I. Vardavas, Filippos T. Filippidis

**Affiliations:** 1 Department of Primary Care and Public Health, School of Public Health, Imperial College London, London, UK; 2 Clinic of Social and Family Medicine, School of Medicine, University of Crete, Heraklion, Greece

## Abstract

Data were analysed from the 2014 Special Eurobarometer for Tobacco survey. We estimated self-rated importance of various factors in the choice of both tobacco and electronic cigarettes (e-cigarettes) among tobacco smokers who had ever used an e-cigarette. Among ever users of tobacco and e-cigarettes (*N* = 2430), taste (39.4%), price (39.2%) and amount of nicotine (27.3%) were the most commonly cited reasons for choosing their brand of e-cigarettes. Those aged 15–24 were more likely to cite external packaging [adjusted prevalence ratio (aPR = 2.06, 95% CI 1.00–4.23)] and design features (aPR = 1.99, 1.20–3.29) as important. As further legislation is debated and enacted enhanced regulation of price, design and marketing features of e-cigarettes may help to reduce the appeal of e-cigarettes.

## Introduction

The use of electronic cigarettes (e-cigarettes) has become more common in recent years, driven in part by their increased availability.^1^ There is emerging data on the characteristics of e-cigarette experimenters; disparities have been noted across socio-demographic characteristics.^2^^,^^3^ There is a possibility that the use of design, manufacture, or marketing strategies banned for conventional tobacco, such as multiple flavours, or advertising strategies such as packaging may be used to attract the youth market. E-cigarettes are often presented as a more economical and healthier alternative to tobacco smoking, although the degree to which this is driving use is unknown.^4^ This paper examines the factors influencing both tobacco and e-cigarette choice among participants who have used both e-cigarettes and cigarettes in their lifetime in the European Union (EU).

## Methods

### Data source

We analysed data from wave 82.4 of the Eurobarometer survey of 28 EU countries in November–December 2014, collected and funded by the European Commission.^5^ The survey uses a multi-stage sampling design, with primary sampling units proportional to population size, to collect data from a representative sample (*n* = 27 801) of the EU population aged ≥15 years, using computer assisted face-to-face interviews. Although response rates are not released by Eurobarometer, population weighting is applied to the data based on age, gender and area of residence, resulting in representative samples. The data are freely available to download.

### Measures

#### E-cigarette use

E-cigarette use was assessed with the question ‘Regarding the use of electronic cigarettes or any similar electronic devices (e-shisha, e-pipe), which of the following statements applies to you?’. Responses were: ‘You currently use electronic cigarettes or similar electronic devices (e.g. e-shisha, e-pipe)’; ‘You used them in the past, but no longer use them’; ‘You tried them in the past but no longer use them’; ‘You have never used them’; and ‘Don’t know’. Responses other than ‘You have never used them’ or ‘Don’t know’ were classified as ‘ever users’.

#### Tobacco smoking

Smoking status was assessed with the question ‘Regarding smoking cigarettes, cigars or a pipe, which of the following applies to you?’ Those who selected the response ‘You currently smoke’ or ‘You used to smoke but you have stopped’ were considered ‘ever-smokers’.

#### Factors influencing the choice of cigarettes and e-cigarettes

Participants who had ever used e-cigarettes were asked ‘What are the factors you consider important in your choice of electronic cigarette or any similar device (e-shisha, e-pipe)?’ Respondents could choose multiple responses between ‘price’; ‘packaging’; ‘flavour’; ‘brand’; ‘type of electronic cigarette (disposable, rechargeable with a cartridge, refillable with liquid)’; ‘amount of nicotine’; ‘design or shape of the electronic cigarette or any similar device and its case’; and ‘marketed health claims’.

Similarly, all ever-smokers were asked ‘How important is or was each of the following factors in your choice of brand of cigarettes? The price; the packaging; the taste of tobacco; the specific brand; the specific tastes such as menthol, spicy, fruity or sweet; the levels of tar, nicotine and carbon monoxide; the design or shape of the cigarette (e.g. slim, colour, capsule)’. Response options were dichotomized as important (‘very important’; ‘fairly important’); and not important (‘not very important’; ‘not at all important’). Both the ‘taste of tobacco’ and specific tastes were grouped together under the label of ‘taste’.

#### Socio-demographic characteristics

Data were collected on participants’ age (15–24; 25–39; 40–54; and ≥55 years), gender (male; female), age at which they stopped full-time education (≤15; 16–19 and ≥20 years old) and whether they have had difficulties in paying bills during the last 12 months (categorized as almost never/never; and from time to time/most of the time).

### Statistical analysis

Analyses for this paper were restricted to respondents who had used both e-cigarettes and tobacco products in their lifetime (*n* = 2430), as they were asked for the factors influencing the choice of both products.

Logistic regression models assessed socio-demographic characteristics of factors influencing choice of e-cigarettes. These models also assessed the relationship between identifying similar factors as important for both e-cigarettes and tobacco products. Analyses used survey weights provided in the official Eurobarometer dataset to ensure that estimates are representative across the population, and results are presented as adjusted prevalence ratios (aPR).

## Results

A total of 2712 individuals had ever used e-cigarettes and, among them, 2430 (89.6%) were ever-tobacco smokers. Among this group taste/flavours and price were the two most commonly reported factors influencing the choice of both tobacco (91.1 and 73.3%, respectively) and e-cigarettes (39.4 and 39.2%, respectively) (table 1). Health claims for e-cigarettes were not commonly cited as a reason for use (12.3%).

**Table 1:** Socio-demographic variations in factors influencing the choice of tobacco and e-cigarettes among individuals aged ≥15 years who have ever tried both cigarettes and e-cigarettes in the European Union, 2014 (*n* = 2430)

	Price (%)	Packaging (%)	Flavour (%)	Brand (%)	Amount of nicotine (%)	Design (%)	Health claims (%)	Type of e-cigarette (%)
Cigarette choice	73.3	18.7	91.1	56.9	48.2	23.1	N/A	N/A
E-cigarette choice	39.2	3.7	39.4	9.4	27.3	9.8	12.3	21.7
	Price aPR (95% CI)	Packaging aPR (95% CI)	Flavour aPR (95% CI)	Brand aPR (95% CI)	Amount of nicotine aPR (95% CI)	Design aPR (95% CI)	Health claims aPR (95% CI)	Type of e-cigarette aPR (95% CI)
Age group (years)								
55+	Ref	Ref	Ref	Ref	Ref	Ref	Ref	Ref
40–54	1.10 (0.93–1.31)	1.60 (0.81–3.16)	1.06 (0.90–1.24)	1.21 (0.76–1.94)	1.15 (0.95–1.40)	1.81 (1.13–2.90)	1.22 (0.92–1.63)	1.41 (1.09–1.82)
25–39	1.27 (1.08–1.50)	1.04 (0.51–2.13)	1.05 (0.90–1.23)	1.76 (1.14–2.73)	1.06 (0.87–1.29)	1.90 (1.20–3.01)	1.20 (0.90–1.60)	1.47 (1.14–1.89)
15–24	1.36 (1.14–1.63)	2.06 (1.00–4.23)	1.09 (0.91–1.31)	1.81 (1.11–2.95)	1.05 (0.83–1.33)	1.99 (1.20–3.29)	1.28 (0.93–1.75)	1.67 (1.28–2.18)
Gender								
Female	Ref	Ref	Ref	Ref	Ref	Ref	Ref	Ref
Male	0.95 (0.86–1.05)	0.98 (0.65–1.48)	0.99 (0.89–1.10)	1.23 (0.94–1.62)	1.00 (0.88–1.13)	1.30 (1.00–1.68)	0.77 (0.64–0.93)	0.91 (0.78–1.06)
Difficulties paying bills								
Never/almost never	Ref	Ref	Ref	Ref	Ref	Ref	Ref	Ref
From time to time/Most of the time	1.24 (1.11–1.38)	1.05 (0.69–1.61)	1.00 (0.90–1.11)	1.24 (0.94–1.63)	1.17 (1.03–1.34)	1.12 (0.86–1.45)	1.00 (0.83–1.21)	1.06 (0.91–1.23)
Age when stopped full-time education								
Up to 15	Ref	Ref	Ref	Ref	Ref	Ref	Ref	Ref
16–19	0.98 (0.82–1.17)	1.32 (0.58–3.04)	1.00 (0.82–1.23)	0.87 (0.54–1.41)	1.23 (0.94–1.60)	1.09 (0.63–1.91)	0.85 (0.62–1.16)	0.96 (0.73–1.28)
20+	0.90 (0.74–1.08)	0.93 (0.39–2.27)	1.13 (0.92–1.39)	0.79 (0.48–1.31)	1.23 (0.94–1.62)	1.58 (0.90–2.77)	0.82 (0.59–1.14)	0.97 (0.73–1.30)
Similar factor influencing cigarette choice^a^								
Not important	Ref	Ref	Ref	Ref	Ref	Ref	N/A	N/A
Important	2.00 (1.70–2.37)	4.91 (3.20–7.52)	1.77 (1.38–2.28)	2.68 (1.89–3.82)	1.79 (1.54–2.07)	2.02 (1.56–2.61)	N/A	N/A

a ‘Similar factor influencing cigarette choice’ denotes a positive response in the question exploring factors influencing cigarette choice for the equivalent factor. For example, in the outcome ‘price’, the ‘similar factor’ is price of cigarettes, for the outcome ‘packaging’ is packaging of cigarettes etc.

aPR = adjusted prevalence ratio.

Respondents who identified price; packaging; flavour; brand; amount of nicotine; or design as important factors for the choice of cigarettes were more likely to identify the same factor as important for their choice of e-cigarettes, with aPRs ranging from 1.77 for flavour to 4.91 for packaging. Younger respondents were more likely to say that price, packaging, brand, design and type of e-cigarette were important compared with those aged 55 years or older. Price was more important to people who had difficulties paying bills [aPR 1.24 (1.11;1.38)], while design was more important to men and health claims more important to women.

## Discussion

This secondary analysis of a representative sample of the EU population has found that, similar to tobacco, taste and price were the most important factors influencing choice of e-cigarettes, and that health claims were not as commonly cited. Despite the similarities, the criteria for choosing e-cigarettes were not identical to cigarettes, indicating that the approach for understanding this market may require further evaluation. The seeming greater receptivity of younger people to design and packaging features mimics findings from tobacco smoking, and indicates the importance of e-cigarette advertising,^6^ which have been noted to display the similar themes of independence as previous tobacco adverts.^7^

The finding that health claims were not cited as a common factor influencing the choice of e-cigarettes is surprising, as this has been a central part of marketing claims. Additionally, experiments in eliciting preferences have found this to be an important determinant of choice of these products.^8^ However, the majority of the respondents considered e-cigarettes either harmful or were unsure of health effects,^5^ which might partly explain the relatively low importance of health claims.

### Strengths and limitations

The Eurobarometer sample is representative of the EU population aged ≥15 years, therefore results can be generalized to the entire EU population, and comparisons made between different member states. The questions on choice of cigarettes and e-cigarettes had slightly different wording and so comparisons between these should be treated with caution. The closed nature of the questions also means that some possible important elements of choice were not reported. While we considered ever-users of e-cigarettes, we acknowledge that an analysis of regular users would provide more meaningful results; however, the number of regular users was low (∼300), thus limiting the value of such analyses. Finally the cross-sectional nature of this study means that causation cannot be inferred and we support calls for future longitudinal research into this area.^9^

## Conclusions

Many of the factors behind the choice of e-cigarettes are similar to those for tobacco cigarettes, and this data suggests that taste and price are the most important factors influencing experimentation. Young people are more likely to be influenced by design and price of e-cigarettes than older people, which raises concerns regarding the degree to which these should potentially be regulated in the growing e-cigarette market.

## Supplementary data

Supplementary data are available at *EURPUB* online.
